# Therapy-resistant nephrolithiasis following renal artery coil embolization

**DOI:** 10.1186/1471-2490-13-29

**Published:** 2013-06-10

**Authors:** Cédric Poyet, Florian Grubhofer, Matthias Zimmermann, Tullio Sulser, Thomas Hermanns

**Affiliations:** 1 Department of Urology, University Hospital Zürich, University of Zürich, Frauenklinikstrasse 10, 8091, Zürich, Switzerland

**Keywords:** Selective renal embolization, Coil-migration, Nephrolithiasis, Renal bleeding

## Abstract

**Background:**

Transcatheter renal artery embolization is an effective and minimally invasive treatment option for acute renal bleeding. Early post-interventional complications include groin hematoma, incomplete embolization, coil misplacement and coil migration. Late complications are rare and mostly related to coil migration.

**Case presentation:**

A 22-year-old woman with a history of recurrent stone disease and a lumbal meningomyelocele underwent bilateral open pyelolithotomy for bilateral staghorn calculi. Post-operatively, acute hemorrhage of the left kidney occurred and selective arterial coil embolization of a lower pole interlobular renal artery was performed twice.

Four years after this intervention the patient presented with a new 15.4 mm stone in the lower calyx of the left kidney. After two extracorporeal shock wave lithotripsy treatments disintegration of the stone was not detectable. Therefore, flexible ureterorenoscopy was performed and revealed that the stone was adherent to a partially intraluminal metal coil in the lower renal calyx. The intracalyceal part of the coil and the adherent stone were successfully removed using the holmium laser.

**Conclusion:**

Therapy-resistant nephrolithiasis was caused by a migrated metal coil, which was placed four years earlier for the treatment of acute post-operative renal bleeding. Renal coils in close vicinity to the renal pelvis can migrate into the collecting system and trigger renal stone formation. Extracorporeal shock wave lithotripsy seems to be inefficient for these composite stones. Identification of these rare stones is possible during retrograde intrarenal surgery. It also enables immediate stone disintegration and removal of the stone fragments and the intraluminal coil material.

## Background

Renal artery embolization (RAE) was initially developed in the 1970s for symptomatic renal haematuria and palliation for unresectable renal tumours [1]. Nowadays, RAE is a widely used, minimally invasive treatment option for various renal or vascular diseases. The most common indications for RAE are palliation of unresectable renal tumors, treatment of renal angiomyolipomas, renal arterio-venous fistulae, renal artery aneurysms, vascular malformations and life threatening or chronic renal hemorrhage. Furthermore, it is used for infarction of renal tumors prior to nephrectomy or radiofrequency ablation [2]. A variety of embolic agents such as metal coils, particulate or sclerosants agents (liquids, foams) are available. The selection of the embolic material depends on several factors such as vessel size, vascular anatomy and hemodynamics. In the case of acute renal hemorrhage, metal coils are most often used [3].

One possible complication of RAE is migration of these metal coils into the collecting system [4]. Coils that migrated completely into the collecting system have been reported to cause symptomatic ureteric obstruction [5-7]. To the best of our knowledge, stone formation after coil migration has so far only once been described before [8]. We report a case of a partly migrated renal metal coil leading to therapy-resistant nephrolithiasis.

## Case presentation

A 22 year-old woman with a lumbal meningomyelocele presented to our center with bilateral staghorn calculi. The patient was known for her atonic neurogenic bladder and she performed intermittent self-catherization for many years. She suffered from recurrent urinary tract infections with repeated episodes of right-sided pyelonephritis. Due to considerable skeletal deformities the patient was considered uneligible for a percutaneous nephrolithotomy. Therefore, bilateral open pyelolithotomy was performed. It was possible to remove the stones completely without any intra-operative complications. For an unknown reason, a stone analysis was not performed after the operation. Eight days after surgery persistent gross haematuria occurred. Renal angiography demonstrated active bleeding from a left lower-pole interlobular renal artery. During renal angiography RAE was performed using three fibered platinum microcoils (2 mm circle diameter, Boston Scientific Corporation, Watertown, USA). After initial cessation of the bleeding, the patient presented two weeks later with recurrent hematuria and a second RAE procedure of the same interlobular renal artery was performed. Three additional microcoils were placed, and bleeding was controlled. No further hemorrhage occurred.

Four years later, the patient presented again with recurrent right-sided pyelonephritis. Following antibiotic treatment asymptomatic leukocyturia and erythrocyturia persisted. These were attributed to the intermittent self-catherization. Although significant growth of Escherichia coli was repetitively detectable in the urine culture, the patient was only treated when she was symptomatic.

**Figure 1:**
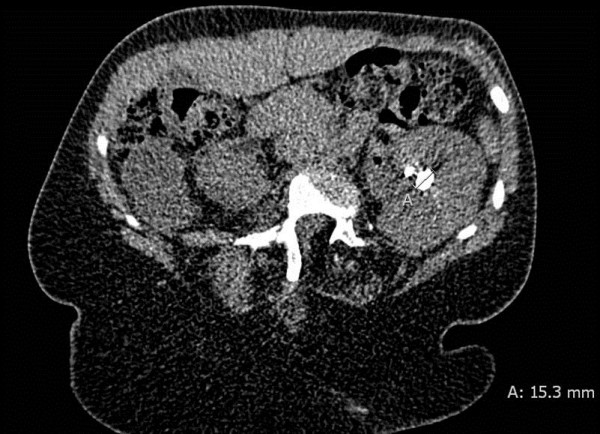
**The pre-operative CT-scan revealed a 15.4 mm stone in the lower calix of the left kidney.** Metal wires in the renal parenchyma were adjacent to the stone. Coiling material in the collecting system or within the stone was not identified.

An abdominal computed tomography (CT) showed an atrophic kidney on the right side and a solitary 15.4 mm stone in the lower renal calyx. The stone was located adjacent to the coiling material placed four years earlier (Figure 1). A radionuclide MAG3 differential renal scan confirmed the diagnosis of an inactive kidney on the right side.

Primarily, an open right-sided nephrectomy was performed. The post-operative course was uneventful and the patient was discharged five days after surgery. Symptomatic urinary infections did not occur anymore after the nephrectomy.

**Figure 2:**
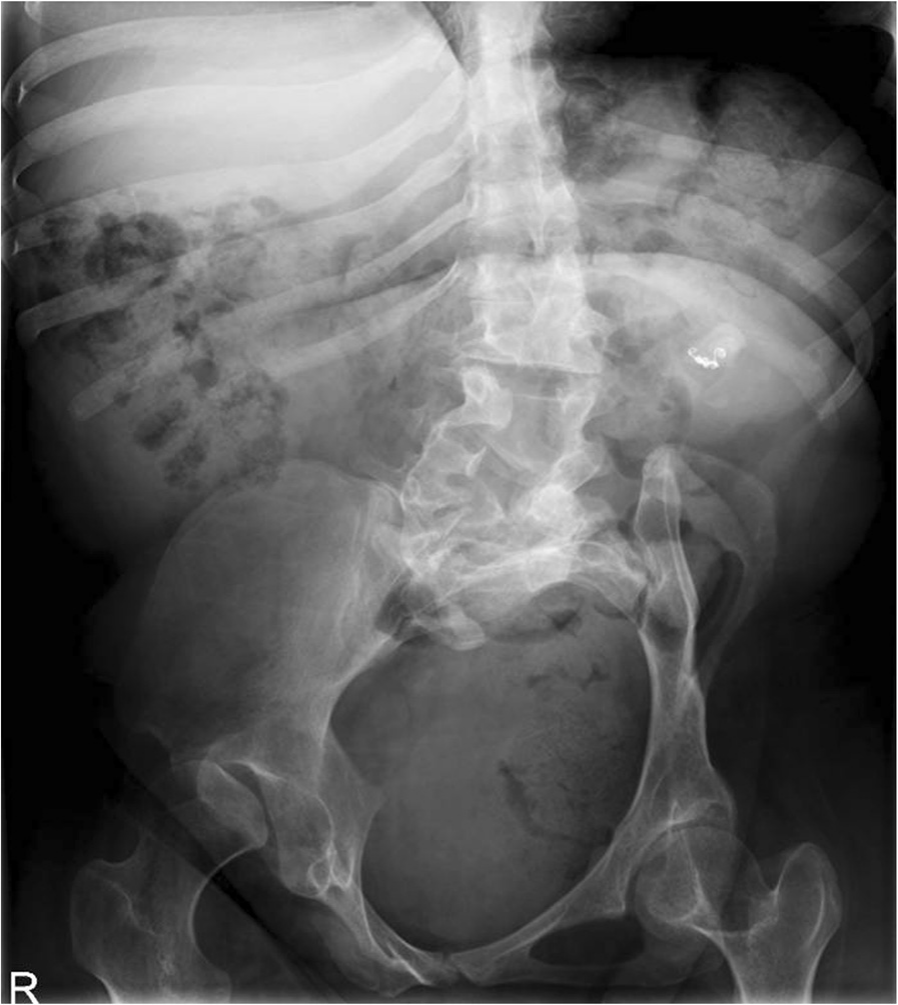
**Plain abdominal film showing the stone after the second ESWL treatment.** The close vicinity of the stone and the coiling material can also be seen.

Three months after the nephrectomy, an extracorporeal shock wave lithotripsy (ESWL) was performed to treat the stone in the left kidney using an electromagnetic Dornier DL50 lithotrypter (Dornier MedTech, Wessling, Germany). A total of 3000 shock waves (16 kV, positive energy of the 5-mm focal area E + 5 mm: 10.1 mJ) were applied. A ureteral stent was inserted to prevent obstructive complications of the left solitary kidney. Follow-up investigations revealed insufficient disintegration of the stone. Thus, two months after the initial treatment, a second ESWL was performed. Six weeks after this ESWL, stone disintegration was still not detectable (Figure 2).

**Figure 3:**
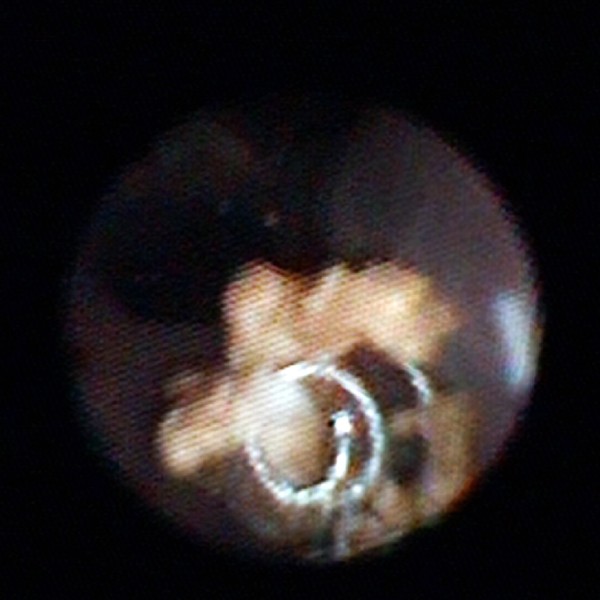
**Endoscopic view during f-URS.** The dislocated coiling material is incorporated into the lower-pole kidney stone.

Subsequently, a flexible ureterorenoscopy (f-URS) was performed. Intra-operatively the solitary stone in the lower calyx was identified. However, the stone was adherent to intraluminal metal coil wires (Figure 3). These metal wires were partly located in the lower calyx and partly in the renal parenchyma. The intraluminal wires were densely integrated into the stone. Despite the two precedent ESWL treatments, the stone appeared almost unaffected. The stone was disintegrated using the holmium laser and removed from the coiling material. The remaining intraluminal coil wires were cut off with the laser at the parenchymal border. Subsequently, the wires and all stone fragments were removed using a dormia basket. At the end of the procedure the renal pelvis was free of stones and coiling material. A ureteral stent was inserted.

After an uneventful peri-operative course, the patient was discharged at the first post-operative day. The ureteral stent was removed two weeks later. X-ray diffraction stone analysis revealed a stone composition of 60% calciumoxalate-monohydrate and 40% apatite. Six months after the procedure, the patient remained stone free as assessed by abdominal ultrasound and plain abdominal film.

## Discussion

A migrated metal coil placed for acute post-operative renal hemorrhage four years earlier induced stone formation in this patient with a history of recurrent nephrolithiasis. The stone and the metal coil wires formed a very robust composite, which resisted two ESWL treatments. Identification and immediate treatment of this composite stone was possible during retrograde intrarenal surgery.

The most common early complication of RAE is the post-infarction syndrome, a combination of post-interventional symptoms such as flank pain, fever, nausea and vomiting. It has been reported to occur in approximately 75% of all cases [4].

Further complications specific to RAE have been reported to occur in 5-10% of the cases [4,9]. Complications can be classified into early and late complications. The most common early complications are groin hematoma, incomplete embolization, coil misplacement and coil migration [4,10]. Late complications are rare and are mainly related to coil migration into the collecting system.

Migration of embolization coils has been reported to occur in less then 2% of all cases [4]. Three cases of symptomatic ureteric obstruction by completely migrated metal coils after up to 1.5 years following RAE have been reported [5-7]. In all cases, the obstructing metal coil had to be removed by ureteroscopy.

It is well known that foreign bodies in the urinary collecting system can induce stone formation [11]. In the present case the foreign body inducing stone formation was a metal coil, which partly migrated into the collecting system. The history of the patient with a myelomeningocele, neurogenic bladder dysfunction, intermittent self-catheterization, recurrent urinary tract infections and right-sided pyelonephritis is suggestive for the development of infection stones. Although the stone analysis after bilateral pyelolithotomy is not available, it is likely that the initial staghorn calculi consisted, at least to some extent, of the typical infection stone material struvite. Patients with musculoskeletal anomalies are more likely to have a stone composition of struvite due to recurrent urinary tract infections [12]. However, the patient in the present case never had a pyelonephritis on the left side and the analysis of the left-sided composite stone revealed a pure calcium-oxalate/apatite composition. It has recently been shown that improved patient care in the last two decades resulted in a significant change of the predominant stone composition in these patients [13]. Nowadays, struvite stones account only for approximately 20% of all stones while stones not related to urinary tract infections such as apatite and calcium oxalate stones are found in approximately 80% of all renal stones in this patient group [13].

The absence of recurrent infections in the left kidney and the presence of a solitary renal calculus which was adherent to the migrated metal coil support the hypothesis that the metal coil was the main trigger for the new onset of left nephrolithiasis.

To the best of our knowledge, stone formation after coil migration as a late complication of RAE has so far only been described once [8]. In this case, however, a renal calculus of 7 mm had formed around a totally migrated metal coil in the collecting system two years after RAE. The stone was treated using a pneumatic lithotripter during semirigid ureterorenoscopy. In contrast to the present case, the intraluminal metal coil was not fixed in the renal parenchyma and ESWL treatment was not performed due to low opacity of the stone.

The metal wire not only induced stone formation but also had an impact on the physical characteristics of the stone and its responsiveness to the ESWL treatment. The intra-luminal coiling material was densely integrated into the stone thereby forming a composite stone. The reinforcing characteristics of composite materials consisting of metal wires and stone or concrete (reinforced concrete) are well known and often used in construction for on-ground floors and pavements [14]. The robustness of the composite stone impeded effective disintegration and detachment of stone fragments from the metal wire, which is necessary for stone clearance after ESWL.

The close vicinity of the coils and the stone was detectable on pre-operative radiographic imaging. In retrospect, stone formation around migrated embolization coils could have been suspected earlier, particularly after the first unsuccessful ESWL treatment. However, initially, this option was not taken into consideration and f-URS was only indicated after two ESWL treatments. Retrograde intrarenal surgery seems to be the optimal treatment option in this situation. It makes direct visualization and thereby identification of the special composition of the stone possible. Furthermore, stone disintegration and removal can be combined with removal of the stone inducing foreign body.

The combination of several risk factors including a solitary kidney, recurrent stone disease and the remaining intra-parenchymal renal coil material, which might also migrate into the collecting system requires this patient to be closely followed in the future.

## Conclusion

Renal metal coils can cause complications years after their initial placement. Stones can form around migrated coiling material. The resulting robust composite stones are to be taken into consideration if stones are identified adjacent to renal coil material. Retrograde intrarenal surgery, which enables identification and simultaneous removal of the stones and the coiling material, should be the preferred first-line treatment option.

## Consent

Written informed consent was obtained from the patient for publication of this case report and any accompanying images. A copy of the written consent is available for review by the Series Editor of this journal.

## Abbreviations

CT: Computed tomography; ESWL: Extracorporeal shock wave lithotripsy; f-URS: Flexible ureterorenoscopy; RAE: Renal artery embolization.

## Competing interests

The authors declare that they have no competing interests.

## Authors' contributions

CP drafted the report, contributed to concept, and cared for the patient. FG drafted the report, and approved the final version of the manuscript. TH contributed to concept and design and made relevant corrections. MZ and TS cared for the patient and approved the manuscript. All authors read and approved the final manuscript.

## Pre-publication history

The pre-publication history for this paper can be accessed here:

http://www.biomedcentral.com/1471-2490/13/29/prepub
